# Acute Intestinal Obstruction as an Initial Presentation of Mesenteric Venous Thrombosis

**DOI:** 10.7759/cureus.15652

**Published:** 2021-06-14

**Authors:** Pradip Vekariya, Dharanesh Daneti, Kuppusamy Senthamizhselvan, Sathasivam Sureshkumar, Pazhanivel Mohan

**Affiliations:** 1 Medical Gastroenterology, Jawaharlal Institute of Postgraduate Medical Education and Research, Puducherry, IND; 2 General Surgery, Jawaharlal Institute of Postgraduate Medical Education and Research, Puducherry, IND

**Keywords:** abdominal pain, anastomosis, anticoagulants, intestinal obstruction, mesenteric ischemia

## Abstract

Intestinal ischemia commonly occurs after arterial thrombosis or embolism. Thrombosis of the mesenteric vein accounts for less than 10% of cases of intestinal ischemia. Superior mesenteric vein thrombosis (SMVT) in its chronic form is less culpable to produce intestinal ischemia as it forms sufficient collateral drainage. Intestinal obstruction due to mesenteric venous thrombosis is rare, and so far, only 12 cases have been reported. The majority of them had a distinct episode of acute abdominal pain due to ischemia and later developed bowel stricture and intestinal obstruction. Here we report a case of a 44-year-old male who presented with intestinal obstruction as an initial presentation of SMVT. The patient required surgical resection and anastomosis, and he was started on anticoagulation therapy. This case report reiterates the fact that persistent low-grade mesenteric venous ischemia may lead to bowel stricture formation at a later stage. Therefore, etiological workup and early anticoagulant therapy can be useful to improve recurrence.

## Introduction

Mesenteric venous thrombosis (MVT) is an uncommon cause of intestinal ischemia. The usual presentation is abdominal pain out of proportion to abdominal signs. In its severe form, it may lead to perforation peritonitis. Rarely the persistence of ischemia may lead to bowel stricture and intestinal obstruction in a patient with superior mesenteric vein thrombosis (SMVT). Intestinal obstruction in SMVT results from persistent ischemia and presents in the later part of the clinical course [1]. The majority of previously reported cases of intestinal obstruction due to SMVT had two stages of presentations. The first stage of bowel ischemia presented with abdomen pain, while later on, they developed bowel stricture and intestinal obstruction [2-6]. Here we report a case of mesenteric thrombosis, with intestinal obstruction as an initial presentation.

## Case presentation

**Figure 1:**
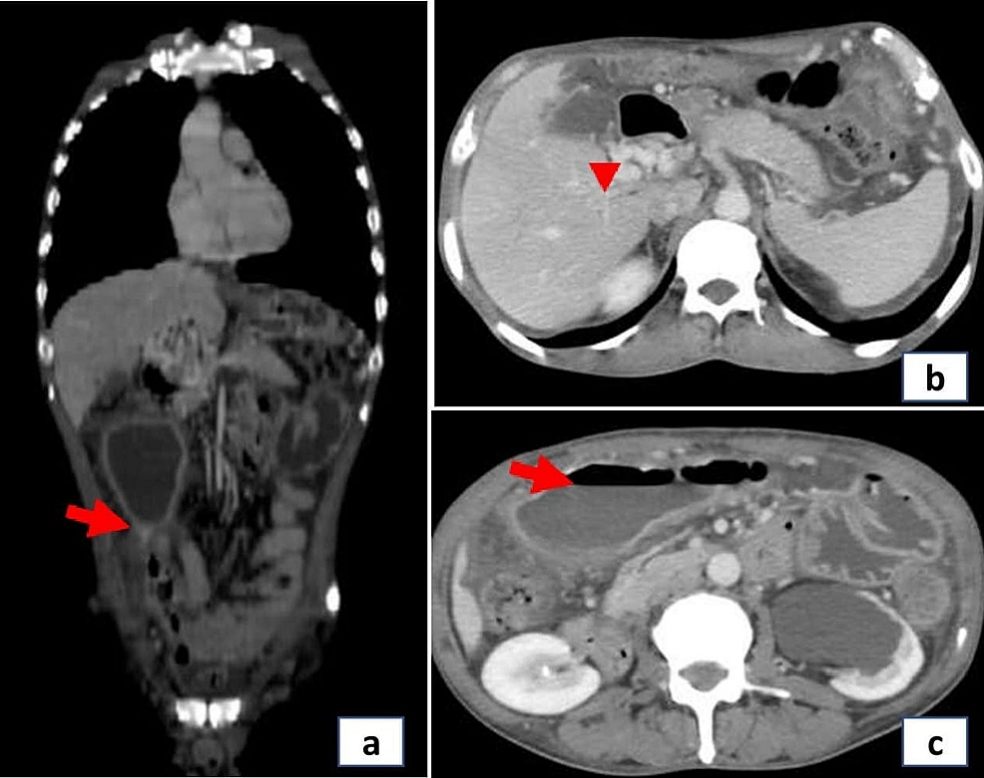
Contrast-enhanced CT abdomen images: a) arrow pointing to a stricture in proximal jejunum, b) arrow head pointing multiple collaterals in the portomesenteric confluence and superior mesenteric vein, c) arrow pointing to upstream dilatation of the bowel with air fluid levels

**Figure 2:**
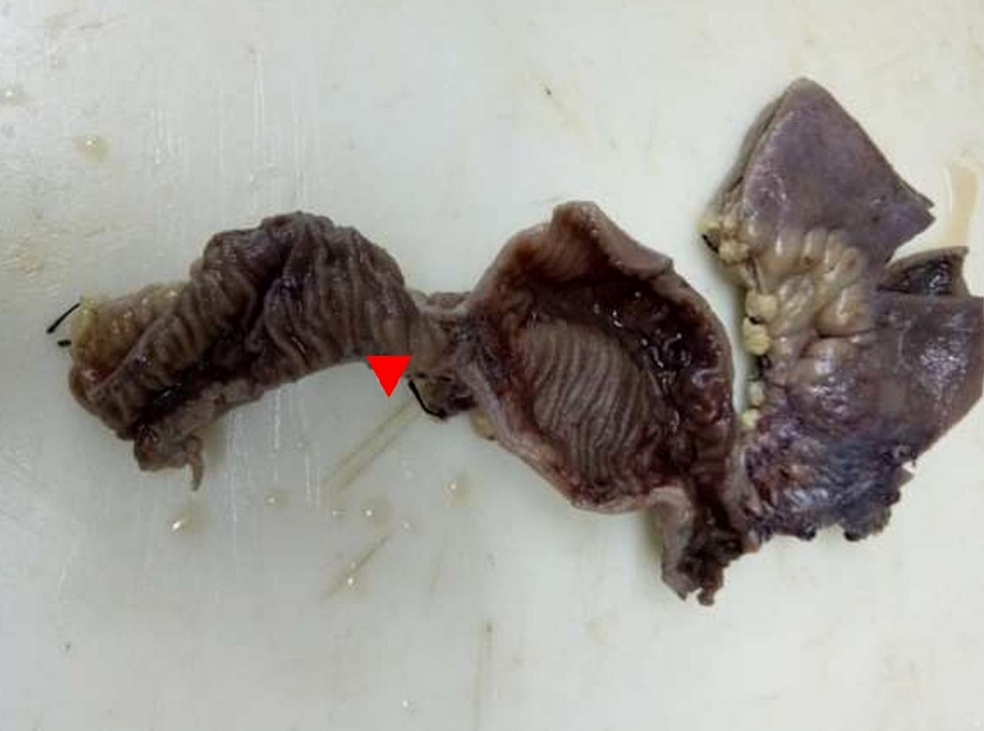
Gross resected specimen – arrow head pointing to a stricture with dilatation of the proximal bowel

**Figure 3:**
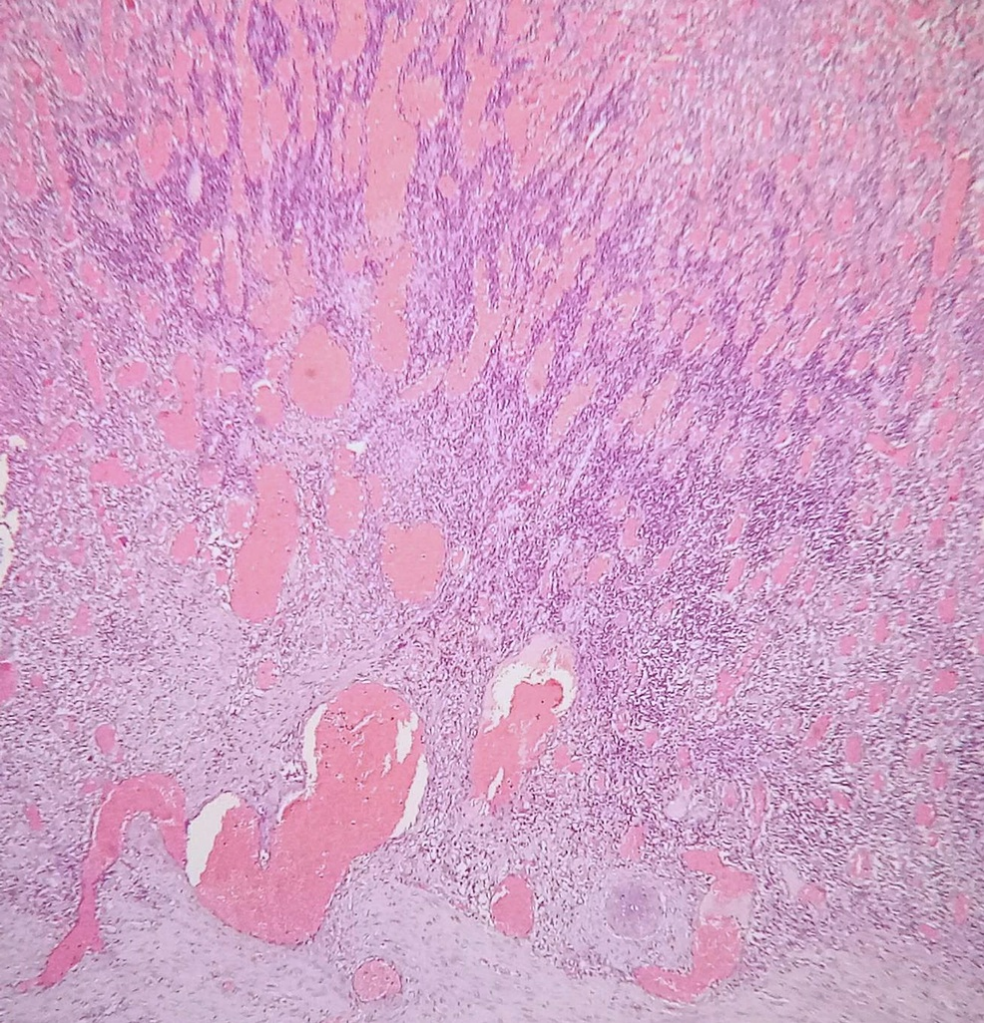
Photomicrograph of histopathology of the resected bowel showing thrombosis of the blood vessels and nonspecific inflammation (hematoxylin and eosin stain, 4X)

A 44-year-old male presented to the emergency department with worsening periumbilical pain and bilious vomiting for four days. He did not have a fever, diarrhea, or gastrointestinal bleed, and his past medical history was unremarkable. He was neither an alcoholic nor a smoker. There was no history of inherited thrombophilic disorders in any of the family members. On examination, the patient had tachycardia and tachypnea. Abdominal examination showed a distended gaseous abdomen with mild diffuse tenderness. X-ray abdomen erect revealed dilated bowel loops with multiple air-fluid levels. His blood sugar, amylase, renal, and liver function tests were normal. Contrast-enhanced CT (CECT) abdomen showed stricture in proximal jejunum with dilated edematous proximal jejunal loops and thrombosis of portomesenteric confluence with extension into the superior mesenteric vein (SMV), and that was replaced by multiple collaterals (Figure 1). The patient underwent an emergency laparotomy. Intraoperatively, we saw a narrowed segment of jejunum with proximal dilatation. Around 12 cm of jejunum was resected, and jejuno-jejunal anastomosis was done. The gross specimen showed critical stricture with dilated proximal bowel (Figure 2). The postoperative period was uneventful. The patient was started on oral liquid diet and gradually accelerated to a solid diet. Histology of the resected specimen showed thrombosed blood vessels and nonspecific inflammation (Figure 3). The patient was evaluated for inherited thrombophilia, and the reports of which were unyielding. He was started on an oral anticoagulant and is presently doing well on follow-up.

## Discussion

Mesenteric artery embolism and thrombosis are common causes of bowel ischemia. In 1895, it was first reported that bowel ischemia could also result from venous thrombosis [7]. MVT is an uncommon etiology of mesenteric ischemia, and it accounts for fewer than 10% of cases of intestinal ischemia [8]. Clinical manifestations of MVT are variable. The usual course of MVT is insidious compared with the rapid fulminate course of mesenteric arterial occlusion. MVT can be categorized into an acute, sub-acute, and chronic variant based on thrombus formation rapidity. In acute MVT, rapid and complete occlusion of the vein results in insufficient collateral development time, leading to bowel ischemia and rarely perforation peritonitis [5]. While in the chronic form, patients develop collaterals and ensure venous drainage. These collaterals prevent transluminal infarction but may not be sufficient to prevent chronic bowel ischemia [1]. During the healing phase of intestinal ischemia, patients may develop fibrosis at a later date that may progress to intestinal stricture and subsequent intestinal obstruction. Intestinal obstruction usually appears several weeks after the initiation of mesenteric vein thrombosis [3]. Evidence of such ischemia and vein thrombosis has been demonstrated in histopathological examination of previous case reports [9].

Here we have enlisted all the reported cases of MVT with intestinal obstruction in the available English literature till this date (Table 1). All of the mentioned cases except one had two stages of presentation [10]. Initially, patients were diagnosed as MVT for abdominal pain and started on anticoagulation, and later on, they presented with features of intestinal obstruction. While in our case, intestinal obstruction was the initial presentation of SMVT. Our patient did not manifest early in the acute phase, probably due to persistent low-grade bowel ischemia, which ultimately developed intestinal fibrosis, stricture, and obstruction. The clinical presentation of MVT is usually nonspecific; hence, a CECT scan of the abdomen may be required for its diagnosis. CECT has more than 90% sensitivity for the diagnosis of MVT [11]. In addition to venous thrombosis, intestinal ischemia can also be assessed in CECT, and this provides a better look for selecting patients eligible for conservative management. The presence of collaterals in the CT abdomen helps to differentiate acute MVT from chronic MVT. In the absence of local inflammatory causes, an etiological workup for mesenteric vascular thrombosis has paramount importance. Inherited thrombophilic states are the common causes for isolated MVT [8]. In contrast, local causes are more commonly seen with combined mesenteric and portal venous thrombosis [12]. In this patient, the etiology could not be identified despite screening for the commonly encountered thrombophilia states. The management goals in MVT are to prevent bowel infarction, perforation peritonitis, and recurrence of the disease. Anticoagulation therapy early in the course of the disease plays a cardinal role. In acute MVT, emergency surgery is indicated in bowel gangrene and perforation peritonitis, whereas in chronic MVT, surgery is required in patients with intestinal stricture and obstruction. In the presence of inherited thrombophilia, patients require lifelong anticoagulation to avoid further complications.

**Table 1:** List of reported cases of mesenteric venous thrombosis with intestinal obstruction EHPVO, extrahepatic portal vein obstruction; PVT, portal vein thrombosis; IO, intestinal obstruction; APLA, antiphospholipid antibody; NA, not available.

Authors	Year	Age/sex	Presentation	CT/MR finding	Pathology finding	Etiological workup
Eugène et al. [2]	1995	36/M	MVT with bowel ischemia later developed IO	PVT, MVT	Venous thrombosis	Intermittent APLA positive
		46/M	MVT with bowel ischemia later developed IO	NA	Venous thrombosis	Negative
		54/M	MVT with bowel ischemia later developed IO	NA	Nonspecific inflammation	Negative
Lin et al. [4]	2012	29/M	MVT with bowel ischemia later developed IO	MVT and PVT	NA	NA
Yang et al. [3]	2012	64/M	MVT with bowel ischemia later developed IO	SMVT	Areas of ischemic wall infarction	NA
Paraskeva and Akoh [5]	2014	64/M	MVT with bowel ischemia later developed IO	PVT/MVT and small intestine ischemia	Active chronic inflammation	Negative
Franco et al. [10]	2015	79/M	MVT with IO managed conservatively	PVT/MVT and bowel distension	-	Protein S and antithrombin-III deficiency
Chou and Huang [6]	2018	27/M	MVT with bowel ischemia later developed IO	MVT	NA	Negative
Al-Taee et al. [9]	2019	33/F	MVT with bowel ischemia later developed perforation and bezoar-induced IO	Small bowel perforation with MVT	Venous thrombosis	NA
Priyadarshi et al. [1]	2021	41/M	EHPVO with MVT led to chronic IO	EHPVO, jejunal vein thrombosis, and jejunal stricture	NA	NA
		25/M	EHPVO with bowel ischemia later developed IO	EHPVO, SMVT, and jejunal stricture	NA	NA
		24/M	EHPVO with MVT led to chronic IO	EHPVO, jejunal vein thrombosis, and jejunal stricture	Intramural thrombosed venules	NA

## Conclusions

A high index of suspicion is required for early diagnosis and management of mesenteric vascular thrombosis. Persistent low-grade mesenteric venous ischemia may lead to bowel stricture formation. Patients who did not manifest in the acute phase may still require surgical resection later due to intestine stricture. Etiological workup and early anticoagulant therapy are crucial to avoid postoperative recurrence.
